# Science-utility and science-trust associations and how they relate to knowledge about how science works

**DOI:** 10.1371/journal.pone.0260586

**Published:** 2021-12-16

**Authors:** Cornelia Schoor, Astrid Schütz

**Affiliations:** 1 Department of Educational Research, University of Bamberg, Bamberg, Germany; 2 Department of Personality Psychology and Psychological Assessment, University of Bamberg, Bamberg, Germany; York University, CANADA

## Abstract

Knowledge about how science works, trust in scientists, and the perceived utility of science currently appear to be eroding in these times in which “alternative facts” or personal experiences and opinions are used as arguments. Yet, in many situations, it would be beneficial for the individual and all of society if scientific findings were considered in decision-making. For this to happen, people have to trust in scientists and perceive science as useful. Still, in university contexts, it might not be desirable to report negative beliefs about science. In addition, science-utility and science-trust associations may differ from explicit beliefs because associations were learned through the co-occurrence of stimuli rather than being based on propositional reasoning. We developed two IATs to measure science-utility and science-trust associations in university students and tested the psychometric properties and predictive potential of these measures. In a study of 261 university students, the IATs were found to have good psychometric properties and small correlations with their corresponding self-report scales. Science-utility and science-trust associations predicted knowledge about how science works over and above self-reported beliefs. The results suggest that indirect measures are useful for assessing beliefs about science and can be used to predict outcome measures.

## Introduction

On the one hand, the COVID-19 pandemic appears to have made many people value science (again) [at least in Germany: 1], but on the other hand, the divide between those who put their trust in science and consider it useful for decision-making and those who do not has appeared to increase. Scientific findings are easy to find these days, but the same is true for less trustworthy information, misinformation, and disinformation [2]. Adequately understanding the various documents that are available necessitates more than reading comprehension [3, 4], especially with regard to considering the source of information [5, 6] and selecting trustworthy sources. A growing number of people seem to rely on personal beliefs and experiences instead of scientifically established knowledge [7], a trend that is considered characteristic of our current post-truth era [2]. The skeptical attitude toward science has been rising in recent years [e.g., 8]. However, now, the COVID-19 pandemic shows vividly that positive beliefs about science are an asset that we should strive for. For example, people who are informed about scientific findings and trust in these findings are more likely to comply with the rules that have been set forth to contain the pandemic [9]. The present paper aims to assess science-utility and science-trust associations as well as explicit beliefs about science and test their predictive potential.

In order to do so, we relied on two lines of research and combined them. Research on the public’s understanding of science [10] has often focused on trust in science, whereas research on teacher education has used the concept of the perceived utility of science [11–13]. In the present paper, we assumed that these two beliefs are both important for laypeople’s considerations of scientific findings: Trust in science is necessary for laypeople to be able to consider scientific information valid [14, 15]. And even if scientific information is considered valid but not useful, people will probably not base their decisions on it [13, 16, 17]. Thus, both trust in science and the perceived utility of science seem to be beliefs that merit further attention.

It has been argued that a lack of positive beliefs about science is related to a lack of knowledge about science [e.g., 10, 18]. To date, studies addressing this relationship have focused on knowledge about basic scientific facts and have largely neglected other aspects of scientific literacy [19], such as knowledge about scientific methods, even though knowledge about methods may be very important for helping people understand and comply with evidence-based suggestions regarding everyday decisions [see 9, 20]. Positive beliefs about science in turn might help students develop knowledge about scientific methods by motivating them to acquire knowledge and by making the best use of their learning opportunities [see 21].

By contrast, mistrust in science is widespread, and interestingly, it is not restricted to populations with little education. Even university students do not consistently prefer information sources with (scientific) expertise over sources who are reporting on their own personal experiences [13, 22]. For example, Salmeron and colleagues [22] had university, primary, and secondary school students rate on a 4-point Likert scale whether or not a fictitious user of a social question-and-answer forum should follow the advice provided in the forum. Forum contributions varied with regard to expertise (expert vs. pseudonym) and source of information (external source vs. personal experiences). On average, participants recommended following the advice, regardless of expertise, source of information, or the educational level of the participants. Kiemer and Kollar [13] asked student teachers to work on a case and provided them with both scientific and non-scientific sources. They found that student teachers more often selected non-scientific than scientific sources.

However, the occurrence of negative beliefs about science is especially problematic in university students because these students will typically have an increased impact on society as a whole in their later professional lives: They will be more likely than others to occupy leading positions, and they may be perceived as paragons of scientific judgment. This makes university students an especially relevant population when studying beliefs about science.

Simply asking university students about their beliefs about science would provide only part of the picture. Direct measures, such as self-reports, tap into propositional reasoning, that is they are assumed to elicit deliberate validation of beliefs [e.g., 23]. Other measures (often referred to as indirect or implicit measures [see 24]) offer a different approach as they tap into associations between concepts [e.g., 23]. Thus, science-utility and science-trust associations should capture different learning experiences than self-report measures. Consequently, indirect measures often offer predictive power over and above their direct counterpart [25, 26]. In addition, self-reports might be affected by social desirability and self-deception [27, 28], which might be issues when university students are asked about their beliefs about science [see 29, but see 30 for a different view].

With the objectives of assessing beliefs about science while avoiding issues of social desirability and taking advantage of the different processes that indirect measures draw on in comparison with direct measures, with the present project, we aimed to develop and test two indirect measures of associations. As outlined above, we focused on two aspects of beliefs about science: whether scientific findings and scientists are considered trustworthy (trust in science) and whether scientific findings are considered useful for decision-making (perceived utility of science). Specifically, the aim of the present study was twofold. First, we aimed to assess two newly developed indirect measures with regard to their psychometric properties and their relations to corresponding direct measures. Second, we examined whether university students’ science-utility and science-trust associations as well as their explicit beliefs about science are related to their knowledge about how science works. Thus, our work extends prior research in several ways: We used methodological knowledge instead of factual knowledge about science, and we developed measures of science-utility and science-trust associations, which stem from two thus-far unconnected lines of research that we combined for the present study.

### Measuring beliefs, attitudes, and associations

Attitudes can be defined as a tendency to evaluate an object with some degree of favor or disfavor [31, 32]. Attitudes can be inferred from cognitive, affective, and behavioral responses [31]. Cognitive responses are often conceptualized as beliefs, that is, associations between an object and various attributes [31]. Thus, whereas attitudes are considered the association between an object and a valence [33] (i.e., positivity or negativity), beliefs encompass associations of the object with other dimensions, such as utility or trustworthiness. In the following, we use the term “evaluation” to refer to both attitudes and beliefs.

Both attitudes and beliefs have been measured directly with self-report questionnaires but also with more indirect procedures [e.g., 34–36]. Explicitly asking people to report their evaluations has advantages, for example, high face validity and ease of administration, but it also comes with disadvantages. Among the latter are a high susceptibility to social desirability, the inaccessibility of the constructs that are asked about, and (self-)deception [29]. Yet, there is evidence that social desirability might not be a major problem [30]. For example, Axt [30] analyzed measures of racial attitudes that varied in their degree of directness. That is, he compared an implicit association test (IAT) of racial attitudes with self-report measures of explicit attitudes that differed in the extent to which racial attitudes were addressed directly. He argued that there was a trade-off between reducing social desirability issues and measuring construct-irrelevant aspects. In this analysis, the most direct measure of explicit attitudes was best at predicting IAT results, and it maximized the differences between participants of different races. Thus, the extent to which social desirability biases direct measures may be debatable.

Several procedures have been developed for indirectly measuring attitudes and beliefs [e.g., 37]. The basic idea behind these procedures is that they do not ask for explicit evaluations but instead use reaction times to measure associations with the object in question. The best known and most valid indirect measure is the implicit association test [IAT; e.g., 36, 38]. For this test, participants categorize two lists of words: a list of words from the evaluative dimension and a list of words referring to the object of interest. The words are displayed on the computer screen, and categorization is carried out by pressing one of two letters on the keyboard. The response time is measured. Participants are supposed to do the categorization as fast as possible while still giving “correct” answers. In several blocks of trials, both lists are practiced before combinations are tested. An algorithm based on the difference in mean reaction times is used as an indicator of the association between the categories [see 38 for how issues such as false responses and long latencies are dealt with]. Even though the IAT score has been criticized for containing aspects that are not process-specific, such as speed-accuracy trade-offs, it clearly captures valid and construct-specific processes [39, 40] and has been shown to have predictive validity [e.g., 41, 42]. IATs have been used to measure both attitudes and beliefs by combining the target words with either evaluative terms (e.g., good/bad) or another category (e.g., female/male).

Direct and indirect measures of evaluations are typically only weakly to moderately correlated [43, 44] as they capture different facets of a construct. It has also been shown that implicit evaluations can predict outcomes over and above direct measures [25, 26]. For sensitive topics, such as stereotypes or racism, the predictive validity of indirect measures is higher than that of direct measures [25]. Some theories on implicit and explicit evaluations explain these findings by referring to two different psychological processes [e.g., 45, but see 24 for a critical discussion]. Indirectly measuring evaluations should tap into implicit evaluations, whereas direct measuring should tap into explicit evaluations. For example, in their associative-propositional evaluation (APE) model, Gawronski and Bodenhausen [e.g., 23, 46, 47] assume that implicit evaluations are based on the automatic activation of associations, whereas explicit evaluations draw on these activated associations but are shaped by propositional reasoning about the attitude object. Associative processes are “driven by principles of feature matching and spatiotemporal contiguity” [48], that is, the co-occurrence of stimuli is important for learning the association. Propositional processes, by contrast, are guided by principles of cognitive consistency; that is, they are based on propositional information that is considered valid [48]. This encompasses explicit information but also observations from which propositional inferences are drawn [48]. The two types of processes are not independent from each other but can influence each other. Yet, they do not necessarily lead to the same result, which explains the dissociation between implicit and explicit evaluations [43].

With regard to the development of university students’ attitudes toward and beliefs about science, explicit evaluations might primarily be driven by what students hear in their lectures about science. By contrast, implicit evaluations might be driven by what they experience at the university, for example, the atmosphere surrounding science, what they observe, or how they feel when learning about science. Thus, implicit and explicit evaluations are susceptible to different kinds of informational input. For example, Dasgupta and Asgari [49] showed that contextual messages—which consisted of biographical information about famous female leaders in their Study 1 and the proportion of female professors in a real-life college situation in their Study 2—do not impact explicit gender stereotypes about leadership but do impact implicit gender stereotypes. However, research on gender stereotypes in science, technology, engineering, and mathematics (STEM) revealed that female STEM role models can impact girls’ explicit beliefs about math [50].

With regard to attitudes toward and beliefs about science, there has not been much research on indirect measures. Denessen et al. [51] measured explicit attitudes and beliefs about teaching science rather broadly, including motivation scales, and related these evaluations to an IAT that combined pictograms with a positive or negative valence with pictograms on science and technology. They found no significant relationships between explicit evaluations and implicit associations in a sample of nearly 140 Dutch primary school teachers.

However, there has been some research on implicit gender stereotypes with regard to STEM, that is, on implicit associations between STEM and gender. Nosek and colleagues showed that science is more often implicitly associated with males than with females [52]. This implicit stereotype predicted sex differences in the science and math achievement of 8^th^ graders. Related work showed implicit associations between male and math [e.g., 53–55], which were also related to self-efficacy, achievement, and enrollment preferences [e.g., 56]. Moreover, as early as primary school, girls have already appeared to develop a negative attitude toward math [e.g., 57]. A more recent study showed that gender stereotypes have weakened within the last decade, and a more neutral view of gender with regard to science is emerging [58].

### Beliefs about science

There has been a great deal of research on “attitudes” toward science, but a great variety of constructs have been subsumed under this umbrella term [59], most of them being actual beliefs about science. In the present study, we targeted trust in science and the perceived utility of science. In doing so, we utilized two different literatures. Research on the public’s understanding of science [10] often analyzes trust in science. By contrast, the perceived utility of science has been a focus of research on teacher education [11–13] and is often based on expectancy-value approaches [16, 17].

From both theoretical and practical points of view, it is worthwhile to combine these two approaches. We argue that when people encounter information about a scientific finding, both their perceived utility of science and their trust in science may be relevant to whether or not they consider this scientific information in their decisions. First, information has to be judged as valid in order to be considered [see epistemic validation: 60]. Given that in many cases, laypeople cannot decide whether some specific piece of information is valid or not (what to believe), they have to rely on the trustworthiness of the source of this information (who to believe) [14, 15]. In the case of scientific findings, they have to trust science and scientists. Second, information—even information that is considered valid—will only be taken into account for decisions and actions if this specific information or this kind of information is considered useful for the question at hand [13, 16, 17]. We thus assume that both trust in science and the perceived utility of science are related to how people respond to science and thus should be visible in the knowledge they have acquired about how science works. We elaborate on these thoughts below.

#### Trust in science

When confronted with multiple documents on a topic, as is the case when one searches the Internet, for example, readers can come across consistent and complementary information but also conflicting information [61]. In order to solve such a conflict, readers have to either decide what is true [62]. Or if they do not have sufficient knowledge about the question at hand, as is most often the case when people are considering scientific information, they must rely on the trustworthiness of the information, that is, they have to decide who to trust [14, 15]. Consequently, for further reading, readers prefer sources that they consider trustworthy [e.g., 63–66]. Characteristics that determine the perceived trustworthiness of a source are, to name just a few, expertise, integrity, benevolence [67], and readiness to admit mistakes/guilt-proneness [68, 69].

Trust in science can be defined as the assumption that scientists will provide true or valid knowledge [70]. As such, it is a concept that includes trust in both science as an institution and the respective people who do science, that is, the scientists. Since non-experts have only limited opportunities to understand how scientific knowledge is generated, trust in science can be considered a necessary condition for using scientific information as a basis for decision-making [15, 71].

Trust in science has been assessed directly in several public surveys in Europe and the US [e.g., the Swedish VA Barometer: 8, for an overview, see 70, or the German Wissenschaftsbarometer: 72]. In recent years, explicit trust in science has been declining [8]. This decline may have been due to reports on the instrumentalization of science or on science’s dependence on external funding [73], as well as the crisis surrounding the difficulties in replicating research [e.g., 74–76] or the falsification of data [77]. Nevertheless, a German representative study suggested that explicit trust in science has begun to increase again during the COVID-19 pandemic [1].

In public surveys, explicit trust in science is usually measured with one item, for example, “How much do you put your trust in science and research?” [72]. To acquire more reliable data, a psychometrically tested scale for measuring the explicit trust of university students was developed [78]. In that study, explicit trust in science was found to be positively related to the number of years a student had spent in college and to the number of college-level science courses a student had taken. To our knowledge, there is no indirect measure of science-trust association.

#### Perceived utility of science

Utility value is an important precondition for decision-making and actual behavior [see 17, 32, 79]. As a component of the expectancy-value framework of achievement motivation [e.g., 16, 17], utility value has been shown to be related to achievement-related behavior and achievement, for example, in reading [80], in college-level psychology [81] and mathematics [82] courses, and in low-stakes tests [83].

The perceived utility of science for society as a whole or for specific parts of it, such as the economy or health, has been addressed in several studies [e.g., 84, 85]. For example, people who report a higher utility of science for the economy also consider nanotechnology especially beneficial [86]. Yet, the perceived utility of science for individual decision-making (henceforth: utility of science) has been researched only infrequently. It has been studied primarily in the context of teacher education [11–13] where it refers to the value that is ascribed to science and scientific evidence for informing decisions that concern teaching. Still, teachers do not necessarily rely on evidence-based approaches but often use personal theories or experiences as a benchmark [12, 87, 88, see also 89]. The relevance of perceived utility is underscored by findings such as that the perceived utility of science is associated with teacher-education students’ motivation to study theory-based coursework as opposed to practical coursework [87], with the quality of their evidence-based reasoning [11], and with their selection of scientific information sources [13]. Moreover, the perceived utility of science has been found to be associated with teachers’ use of research-based information [90, 91].

Perceived utility of science has been assessed only directly, by means of questionnaires such as the one used in the present study (see S1 Appendix). To our knowledge, there is no indirect measure of science-utility association.

### Beliefs about science and knowledge about how science works

In the context of the public’s understanding of science, it has been suggested that a lack of positive attitudes toward science and positive beliefs about science are related to a lack of knowledge [e.g., 10, 18]. Likewise, it has been found that more positive beliefs about science are related to better knowledge about science [e.g., 78, 85, 92]. More positive beliefs can lead to more knowledge, for example, because people more often choose situations in which they can learn about science [see 21] or because they benefit more from learning opportunities than their peers with less positive beliefs about science.

Whereas most prior research has conceptualized knowledge as knowledge about scientific facts or content knowledge, this is only one component of scientific literacy. Other aspects are knowledge about how science works or methodological knowledge and knowledge about the impact of science on society [19, 93, 94, see also 95]. In the context of decision-making in one’s professional, personal, or societal life, it is not only prior content knowledge (i.e., scientific facts about the topic) but also knowledge about how science works that seems to be important in situations in which a person has no or not enough prior content knowledge [see 96]—which is probably the case for most socio-scientific issues.

Whereas the relation between beliefs about science and knowledge about scientific facts has been examined quite a bit, there is very little research on the relation between beliefs about science and knowledge about how it works [92]—even though it is plausible that beliefs shape people’s motivation to acquire knowledge in a given area. In the few existing studies, it was found that laypeople’s knowledge about how science works was related to their level of education [92], their science-friendly beliefs [92], their perception of the uncertainty of scientific evidence [92], and their perception of the benefits of nanotechnology [86]. To the best of our knowledge, there are no studies on the relation between science-utility and science-trust associations and knowledge about science with respect to scientific facts or methodological knowledge.

### The present study

The aim of the present study was to analyze two newly developed indirect measures of science-utility and science-trust associations and to relate them to knowledge about how science works by predicting knowledge from these indirect measures in addition to direct ones.

Specifically, we posed the following research questions and hypotheses:

1. What are the science-utility and science-trust associations as well as explicit beliefs about science in a sample of university students? As an exploratory analysis, we also investigated whether there are gender differences in these measures. On the basis of Nadelson et al. [78], who found a mean trust in science of 3.53 on a 5-point Likert scale, we expected a mean trust in science slightly above the scale midpoint. Due to gender stereotypes and related self-stereotyping, we expected gender differences in favor of men that would be more pronounced in the direct measures because these are affected by biases and distortions more often than indirect measures are [e.g., 97]—in our case, conformity with gender stereotypes [e.g., 98].
2. What are the psychometric properties of the science-utility and science-trust measures? We aimed to test the internal consistencies of the indirect measures and the relationships between the indirect measures with the corresponding direct measures. On the basis of prior research [e.g., 43, 44, 90], we expected moderately positive relationships between the indirect and direct measures.
3. How do science-utility and science-trust associations as well as explicit beliefs about science relate to knowledge about how science works? Do science-utility and science-trust associations incrementally predict knowledge about how science works beyond the corresponding explicit beliefs? On the basis of prior research on other beliefs and their ability to incrementally predict outcome variables [e.g., 42], we expected a small to medium-sized incremental effect.

## Method

The research was approved by the local ethics committee of the University of Bamberg. The participants provided written informed consent. No minors were involved.

### Sample

A total of 261 German university students took part in the study. They were 18 to 68 years old (*M* = 22.47, Median = 21, *SD* = 4.71, 78.2% female). (In Germany, it is not unusual for senior citizens to study at the university. The 68-year-old person’s data were checked with regard to whether outliers were present. This was not the case, and thus, we did not exclude the person’s data). The participants were mainly enrolled in psychology (28.4%), teacher education (18.4%), educational science (15.7%), communication sciences (11.1%), or other social sciences (14.9%), which explains the large proportion of women in the sample (see also the S1 Appendix). 66 participants were in a Master’s program, 141 in a Bachelor’s program, and 48 in a teacher education program, most of which are not part of the bachelor/master system but end in a state examination. The study took place from October to November 2019.

We planned to have a sample size of 260 because a power analysis [with GPower 3.1: 99] showed that this sample size would be large enough to detect a small to medium-sized effect (correlation of *r* = .20) with a power of 95%. The participants were recruited via their university courses, university e-mail lists, as well as posters and flyers on campus. They received 10€ for about one hour of testing time. The present study was approved by the local ethics committee.

### Design

We used a correlational design. All participants worked on measures of explicit beliefs about science and science-utility and science-trust associations (predictors) before completing the test of knowledge about how science works (criterion). To control for potential order effects, the order of direct and indirect measures was balanced across participants.

### Procedure

Participants were tested individually on a computer in a room at the university. After being welcomed by the experimenter, they were placed at the computer and asked to work on the study independently. The experimenter remained in the room but was out of the participants’ line of sight.

After providing written informed consent, the participants completed the measures on beliefs, the order of which was balanced across participants. Then, epistemic beliefs and knowledge about how science works were assessed. Due to unsatisfactory psychometric characteristics of the epistemic beliefs scales—more precisely, unsatisfactory fit indices in a confirmatory factor analysis—they were not included in the analyses used in the present study. Finally, participants were asked for demographics. In addition, they took a pretest for another study. When they had finished, the experimenter thanked the participants, and they received their monetary compensation. A list of all measured variables is available in the S1 Appendix.

### Material and instruments

#### Self-report: Utility of science and utility of personal experiences

The direct measure of utility of science was a questionnaire consisting of two scales with four items each to measure the perceived utility of science and the perceived utility of personal experiences for one’s own decisions, which we had already used in another as yet unpublished study. The questionnaire is based on two scales by Kiemer and Kollar [11, 13], which were designed to measure teacher education students’ perceived utility of empirical educational science and their personal experiences for their teaching decisions. The items were rephrased to refer to science in general and to individual decisions, for example, in the health context. A sample item from the utility of science scale is “Scientific knowledge is useful for individual decisions.” A sample item from the utility of personal experiences scale is “Individual decisions should mainly be based on one’s own experiences or those of others.” The items were answered on a 5-point Likert scale. A confirmatory factor analysis (CFA) showed an acceptable fit (χ^2^ = 48.64, df = 19, *p* < .001; RMSEA = .08; CFI = .94; SRMR = .07). The internal consistencies of the scales were calculated as ω total in the R package *psych* [100]. McDonald’s ω is a measure of internal consistency, which is similar to Cronbach’s α but accounts for many problems with the latter [101]. In the present study, the internal consistencies of the scales were acceptable with McDonald’s ω values of .79 for the utility of science and .76 for the utility of personal experiences.

#### Self-report: Trust in science

The direct measure that we used to assess explicit trust in science was a German translation of the questionnaire on trust in science by Nadelson and colleagues [78]. It used a 5-point Likert scale. All items that had been translated from the original scale had been pretested, and on the basis of the pretest results (i.e., factor loadings < |.4|), we included only Items 5, 7, 9, 10, 11, 12, 14, and 15 from the original questionnaire. A sample item is “We can trust scientists to share their discoveries even when they don’t like their findings” [78]. As in our previous study, the short scale had a good internal consistency (McDonald’s ω = .82), and when we allowed two items with almost identical wordings (10 and 11) to correlate, the fit of the CFA was good (χ^2^ = 37.70, df = 19, p = .007, RMSEA = .06, CFI = .96, SRMR = .04).

#### Science-utility and science-trust associations

As indirect measures of science-utility and science-trust associations, two implicit association tests (IATs) were developed. For both IATs, the classical IAT design was used to contrast science with opinion. The evaluation dimension was different for the two IATs in order to mirror the association of science with either utility or trustworthiness. For the IAT on utility, words had to be classified into useful versus useless. For the IAT on trust with the evaluation dimension trustworthiness, words had to be classified into trustworthy versus untrustworthy. All the words used in the IATs are presented in Table 1.

**Table 1:** Words used in the IATs.

IAT	Category	German original	English translation
both	science	Experiment	experiment
		Forschung	research
		Statistik	statistics
		Labor	lab
		Versuchsanordnung	experimental arrangement
	opinion	Ansichtssache	matter of opinion
		Meinung	opinion
		Empfinden	sensation
		Überzeugung	conviction
		Eindruck	impression
utility	useful	Nützlich	useful
		brauchbar	usable
		wertvoll	valuable
		sinnvoll	meaningful
		nutzbringend	beneficial
	useless	unnütz	unncessary
		überflüssig	needless
		zwecklos	futile
		nutzlos	useless
		sinnlos	meaningless
trustworthiness	trustworthy	vertrauenswürdig	trustworthy
		glaubwürdig	credible
		ehrlich	honest
		aufrichtig	sincere
		verlässlich	reliable
		zuverlässig	authentic
		unverfälscht	unbiased
	untrustworthy	zweifelhaft	dubious
		unglaubwürdig	implausible
		unehrlich	unfrank
		unredlich	dishonest
		fragwürdig	questionable
		verlogen	false
		fadenscheinig	specious

For both IATs, a score was computed with the improved scoring algorithm [38]. In this score, 0 indicates that science has a balanced association with utility and inutility or trustworthiness and untrustworthiness. Positive values indicate a stronger association of science with utility or trustworthiness, whereas negative values indicate a stronger association of science with inutility or untrustworthiness. The IATs were implemented with the Inquisit software by Millisecond. Following the recommended procedure [38], error trials were handled by requiring participants to correct their responses, and trials with latencies > 10,000 ms were excluded. Greenwald et al. [38] also recommended that participants for whom more than 10% of their trials had a latency of less than 300 ms should be excluded, but this did not apply to any of the participants in the present study.

#### Knowledge about how science works

Knowledge about how science works was assessed with a scenario-based test, which was based on the test by Retzbach and colleagues [92] and had been used in our previous study. The test included 9 scenarios that scientists may find themselves in. The gender of the scenario scientist was alternated across scenarios. The topics of the scenarios were research design, probability, double-blind procedure, kinds of studies (meta-analysis), significance, falsification principle, peer review, operationalization, and generalizability. As such, the test covered methodological questions about the natural or social sciences, which are the disciplines that are most relevant for socio-scientific issues. It was *explicitly not* the aim of the test to assess methodological knowledge from all possible scientific disciplines or specialized knowledge, such as knowledge about specific methods of analysis (e.g., ANOVAs or *t* tests), but to cover methodological knowledge that is part of a general knowledge base and relevant for socio-scientific issues. Nevertheless, students from the natural or social sciences might have an advantage on this test because they may have encountered these topics in their studies. For each scenario, the participant was asked to choose the correct option from four alternatives. The number of correctly solved scenarios was summed up, resulting in a maximum score of 9. The original German items and their English translations can be found in the S1 Appendix.

### Data availability and analysis

The data that support the findings of this study have been deposited in the Open Science Framework with the URL https://doi.org/10.17605/OSF.IO/Z6AXP.

The data were analyzed with Mplus 8.6 [102], R 4.0.4 [103], and the package *psych*, version 2.0.12 [100].

## Results

Table 2 presents descriptive statistics for the variables that were included in the study. The zero-order correlations of the manifest variables can be found in Table 3.

**Table 2:** Descriptive statistics for the scales and gender differences.

	Scale range	Range	Internal consistency	M (SD)	M (SD) Women (n = 204)	M (SD) Men (n = 56)	t	df	p
Explicit: utility of science	1–5	1.75–5	.79 ^a^	3.74 (0.68)	3.66 (0.68)	4.04 (0.59)	-4.16	99.65	< .001
Explicit: utility of personal experiences	1–5	2–5	.76 ^a^	3.93 (0.61)	3.93 (0.61)	3.91 (0.63)	0.25	86.29	.807
Explicit: trust in science	1–5	1–4.88	.82 ^a^	3.46 (0.6)	3.40 (0.56)	3.69 (0.69)	-2.85	75.52	.006
IAT utility of science	-2–2	-1–1.25	.86	0.15 (0.43)	0.12 (0.44)	0.24 (0.39)	-1.94	96.95	.055
IAT trust in science	-2–2	-1.13–1.33	.90	0.16 (0.46)	0.13 (0.45)	0.27 (0.49)	-1.84	83.10	.070
Knowledge about how science works	0–9	0–9	--	5.47 (1.82)	5.41 (1.81)	5.71 (1.87)	-1.08	85.37	.282

*Note*. ^a^ McDonald’s ω.

**Table 3:** Zero-order correlations of the manifest variables.

	(2)	(3)	(4)	(5)	(6)
(1) explicit: utility of science	.17**	-.01	.17**	.14*	.18**
(2) explicit: trust in science		.11	.07	.10	.09
(3) explicit: utility of personal experiences			-.00	.02	-.01
(4) IAT utility of science				.54***	.15*
(5) IAT trust in science					.23***
(6) knowledge about how science works					

*Note*. * *p* < .05,

** *p* < .01,

*** *p* < .001.

### Science-utility and science-trust associations as well as explicit beliefs about science in the sample and gender differences

In order to analyze science-utility and science-trust associations as well as explicit beliefs of the sample (RQ 1), a *t* test was calculated in R on the manifest d values of the two IATs to test them against 0, and on the mean scores of the self-report scales to test them against the scale midpoint of 3. As can be seen in Table 2, there were slightly positive associations of science with utility (*M* = 0.15) and trustworthiness (*M* = 0.16). Both were significantly different from zero (utility: *t* = 5.50, df = 260; *p* < .001; trustworthiness: *t* = 5.67, df = 260; *p* < .001). Regarding explicit beliefs, the means for utility of science (*M* = 3.74) and trust in science (*M* = 3.46) were also slightly above the scale midpoint of 3. Both were significantly different from 3 (utility of science: *t* = 17.55, df = 260; *p* < .001; trust in science: *t* = 12.53, df = 260; *p* < .001).

The differences in science-utility and science-trust associations as well as explicit beliefs about science by gender are also presented in Table 2. Gender differences were analyzed by means of *t* tests in R. There was a significant gender difference such that men had more positive explicit beliefs about science than women. For science-utility and science-trust associations, there were no significant gender differences. In order to analyze whether gender effects differ between direct and indirect measures, the correlation of gender and explicit utility was compared to the correlation of gender and the science-utility association. The same was done for gender and its correlation with explicit trust and the science-trust association. A Steiger test for dependent correlations showed in both cases that the gender effects in beliefs did not differ significantly across the mode of measurement (utility: *t* = -1.53, *p* = .128, trust: *t* = -0.97, *p* = .335).

### Psychometric properties of indirect measures: Internal consistencies and the relationship between science-utility and science-trust associations and explicit beliefs about science

In order to calculate the internal consistencies of the IATs, the critical IAT trials were divided into four quarters, and D scores were built for each of these quarters, following the procedure outlined by Buttrick and colleagues [104]. The internal consistencies were calculated on the basis of these four D scores. The internal consistencies were very good for the IAT on the utility of science (ω = .86) and for the IAT on the trustworthiness of science (ω = .90). These values are in the upper tail of the typical range for the IAT [26, 105].

In order to assess the relationships between the indirect and direct measures, two confirmatory factor analyses were calculated in Mplus, one for the measures of trust and one for the measures of utility. For the IATs, the four D scores were used as indicators of each latent variable. Regarding the relationships between science-utility and science-trust associations and their corresponding explicit beliefs, there was a moderately positive correlation for utility of science but not for trust in science. In the model including the science-utility association and explicit utility of science (χ^2^ = 11. 26, df = 19, *p* = .915, RMSEA = .00, CFI = 1.00, SRMR = .02), there was a latent correlation between the two measures of .23 (*p* = .002). With regard to the model on the science-trust association and explicit trust in science (χ^2^ = 67.80, df = 52, *p* = .070, RMSEA = .03, CFI = .98, SRMR = .04), the latent correlation of .12 (*p* = .109) was not significantly different from zero. Again, these findings dovetail with previous evidence [37, 43].

### Relationship of knowledge about how science works with beliefs and associations

To analyze the relationship between knowledge and beliefs (RQ 3), several structural equational models were calculated in Mplus (see Table 4). Knowledge was included as a manifest variable, whereas beliefs and associations were modeled as latent variables. Beliefs and associations were allowed to correlate in all models. In contrast to manifest regression analyses, structural equational models can account for measurement error [104].

**Table 4:** Standardized coefficients for beliefs and associations predicting knowledge about how science works in Models 1–9.

	Knowledge about how science works								
	Model 1	Model 2	Model 3	Model 4	Model 5	Model 6	Model 7	Model 8	Model 9
Predictor	Standardized coefficients								
Explicit: utility of science	.21**		.17*				.20**		.16*
IAT utility of science		.18**	.14*					.05	.03
Explicit: trust in science				.09		.07	.05		.04
IAT trust in science					.23***	.22***		.20*	.18
Goodness of fit									
χ^2^	2.92	4.31	12.59	47.53	5.65	79.73	87.84	25.34	195.11
df	5	5	25	26	5	62	62	25	179
*p*	.713	.506	.981	.006	.342	.064	.017	.443	.194
RMSEA	.00	.00	.00	.06	.02	.03	.04	.01	.02
CFI	1.00	1.00	1.00	.95	1.00	.98	.96	1.00	.99
SRMR	.01	.02	.02	.04	.02	.04	.05	.02	.04

*Note*. * *p* < .05,

** *p* < .01,

*** *p* < .001. All predictors in a model were allowed to correlate.

First, only the direct measure of utility of science was entered (Model 1). Then, only the science-utility association was tested as a predictor (Model 2). In a third model, both explicit utility of science and the science-utility association were included. In this model, the science-utility association was able to predict knowledge about how science works (β = .14, *p* = .034) beyond the contribution of explicit utility of science (β = .17, *p* = .014).

In an analogous way, three models were calculated for trust in science (Models 4–6). The science-trust association predicted knowledge (β = .22, *p* = .000) beyond the contribution of explicit trust in science (β = .07, *p* = .329) in Model 6. However, explicit trust in science did not significantly predict knowledge about how science works even as a single predictor in Model 4 (β = .09, *p* = .179).

In a third series of models (7–9), both utility and trust were included. In Model 7, which included explicit utility and trust, only explicit utility of science significantly predicted knowledge about how science works (β = .20, *p* = .006). This was also true when all four measures (explicit utility, explicit trust, science-utility association, science-trust association) were included (β = .16, *p* = .031).

## Discussion

The present study had two main aims. First, we aimed to analyze two newly developed indirect measures of associations, that is, an IAT for the perceived utility of science and an IAT for trust in science, concerning their psychometric properties, including their relationships with corresponding direct measures. Second, we aimed to test how science-utility and science-trust associations as well as explicit beliefs about science relate to knowledge about how science works and whether science-utility and science-trust associations incrementally predict knowledge. We found very good internal consistencies for the two IATs of .86 for the utility of science and .90 for the trustworthiness of science, and small correlations between the indirect and the corresponding direct measures. The science-utility association predicted knowledge over and above explicit utility. Whereas explicit trust did not significantly predict knowledge, the science-trust association did. The results are discussed more in-depth in the following.

For science-utility and science-trust associations as well as for both explicit belief measures, we found that the mean score in our sample was slightly and significantly above the scale midpoint. Since the study was not representative, this provides insight only into the present sample. As participation in the study was voluntary and the study had been advertised as a study on beliefs about science, only students with relatively favorable beliefs about science may have decided to participate. Also, students studying disciplines different from the ones represented in our study may have different beliefs about science. Nevertheless, the present findings are well-aligned with Nadelson et al. [78], who found a mean trust in science of 3.53. In our study, the mean for explicit trust in science was 3.46. Whereas we found gender differences in explicit beliefs about science, no such differences were found in science-utility and science-trust associations. Male students in our study reported higher perceived utility of science and more trust in science than female students did. Descriptively, men also had higher values than women in science-utility and science-trust associations; however, this difference was not significant. The present sample was not composed of equal proportions of men and women, and thus, the finding should be interpreted with caution. Moreover, results on gender might be biased by students’ enrollment in different study programs that differ in how they approach the teaching of scientific methods. Thus, male and female students might have had different experiences with science due to a different exposure to science and scientific methods. Bearing in mind these limitations, this finding is in line with many prior studies that found more positive explicit attitudes toward and beliefs about science in men [see 59, 106] and with the only study on implicit associations with science that we are aware of [51], but is called into question by findings on implicit gender stereotypes regarding science [e.g., 52].

The newly developed IATs on the utility of science and trust in science showed good psychometric properties. The internal consistencies were very high and reached the upper tail of the usual range of internal consistencies in IATs [26, 105]. Regarding the relationships between the IATs and their corresponding direct measures, we found a correlation of .23 for utility of science. This value is perfectly in line with the correlation a meta-analysis found between IAT measures and their direct counterparts [rho = .24: 43]. Conversely, the correlation between explicit trust in science and the science-trust association was only .12 and was nonsignificant. Such low correlations have also been observed in previous studies [44, 107]. In the present study, one reason for this finding could be that the alignment between the direct and indirect measures was not perfect. We used the IAT to assess the association between trustworthiness and science. On the self-report questionnaire [a short version of 78], trust in science encompassed both trust in science as a field and trust in the people who do science, that is, the scientists. The latter items focused on the belief that scientists are trustworthy when they write up their reports. Even though these differences sound subtle, it may be interesting to follow up on such a distinction.

Another explanation is based on the assumption that associations and explicit beliefs are based on different psychological processes and different learning experiences [e.g., 23, 46, 47]. Whereas the learning of explicit beliefs requires the learner to evaluate propositional information as valid, the learning of associations is supposed to take place through the co-occurrence of stimuli. The science-trust association may have a stronger emotional component than the science-utility association, which may have a stronger cognitive component. Thus, the learning of the science-utility association may be more closely connected to the learning of the explicit utility of science, whereas the dissociation may be greater for trust in science. However, the cognitive versus emotional charges of these beliefs are speculations that require further empirical examination.

As expected, we found that the indirect measures relate to knowledge about how science works. In fact, the science-utility association predicted knowledge over and above the corresponding direct measure. And whereas explicit trust did not predict knowledge, the science-trust association did. This finding is notable in several ways. First, in contrast to earlier studies, we did not measure content knowledge, but rather, methodological knowledge. Our measure was focused on methodology and principles in natural and social sciences as these disciplines mainly contribute to decision-making in socio-scientific issues, that is, in areas where laypeople have little content knowledge but have to make important everyday decisions—as has been the case during the COVID-19 pandemic. This choice proved reasonable, as this type of knowledge was related to science-utility and science-trust associations as well as explicit utility of science in the present study.

Second, it is interesting that knowledge about how science works was not significantly related to explicit trust in science in our study. As discussed above, the trust in science scale conceptualizes trust in science as trust in the principles of science and in the people doing science. It may be the case that knowledge about how science works is not related to trust in scientists but is instead related to trust in the principles of science. The indirect measure of trust in our study addressed trust in the principles of science, and it was related to knowledge. However, this explanation has to be tested in further studies. Finally, the findings provide evidence for the validity and usefulness of the newly developed indirect measures of science-utility and science-trust associations.

In the present study, two lines of research were combined, findings on trust in science and on the utility of science. The basic idea is that both (a) trust in science and scientists and (b) the perceived utility of science are necessary for laypeople to consider scientific findings in their decision-making, for example, in the COVID-19 pandemic. The present study offers a first step in the direction of a joint framework of these two beliefs. With regard to knowledge about how science works as a predicted outcome, it seems that both trust and utility independently predict knowledge but do not do so incrementally. Yet, since knowledge is related to both trust and utility, the direction of effects could also be the opposite, such that knowledge influences beliefs about science. Moreover, the relation could be reciprocal. In addition, further studies are necessary to determine whether beliefs about science influence actual decision-making.

Obviously, the present study has its limitations. One limitation is the small proportion of men in the sample, which limits the interpretation of the gender effects we found. Moreover, the present sample is not representative, which limits the generalizability of the positive attitudes toward science that we found in the sample. However, these aspects do not necessarily constrain the interpretation of the relation between science-utility and science-trust associations as well as explicit beliefs about science and knowledge about how science works. The cross-sectional nature of the study does not allow us to draw conclusions about the direction of the relationship between associations and beliefs, and knowledge. Both directions seem plausible. Better knowledge about how science works could foster positive beliefs about and associations with science [10, 18, 78, 85, 92], but more positive beliefs about and associations with science might also direct students’ attention to opportunities to learn about how science works, either because students will pay more attention to the information they encounter or because they will enter into more situations in which they can learn about how science works [see 21]. As in other domains [e.g., reading self-concept and reading comprehension: 108], it also seems plausible that there could be a reciprocal relationship between attitudes and knowledge. To address this question, longitudinal or experimental studies are necessary. Moreover, it is also possible that a third variable, for example, scientific education or exposure to science, influences both knowledge about how science works and beliefs about and associations with science, so that there might not be a direct relationship between the two variables.

Despite these limitations, the present study suggests that science-utility and science-trust associations are constructs that should be considered in future studies. Indirectly measured associations have been shown to be less susceptible to biases and faking than self-reports are [109], draw on different psychological processes than explicit beliefs do [e.g., 23, 46, 47], and as shown in our study, often have incremental validity over explicit beliefs in predicting outcomes [41]. In the present study, the outcome in question was knowledge about how science works. In the future, studies dealing with the question of how people evaluate and consider scientific information when confronted with either single or multiple documents of varying characteristics [63, 110], be it with regard to the COVID-19 pandemic or other topics, might benefit from including not only direct measures of beliefs about science but also science-utility and science-trust associations. Moreover, they might benefit from the simultaneous consideration of trust and utility, since these two beliefs might be intertwined in predicting decisions for which scientific findings could be relevant. From an educational perspective, the present results can contribute to the assessment of indirectly measured associations in addition to explicit beliefs about science. We hope that considering both types of measures will help students more effectively reflect on their beliefs so that they will eventually develop positive science identities [111] and identify with scientific virtues, attitudes, and beliefs.

## Supporting information

S1 Appendix (PDF)
