# Supplementary material for: Science-utility and science-trust associations and how they relate to knowledge about how science works
Source: PLoS One. 2021 Dec 16;16(12):e0260586. doi: 10.1371/journal.pone.0260586 (PMC8675735; doi:10.1371/journal.pone.0260586)
Supplement: S1 Appendix — (PDF) [file pone.0260586.s001.pdf]

S1 Appendix

In this appendix, the following information is provided:

- Variables assessed in the study
- Questionnaire on utility of science and of personal experiences
- Knowledge about how science works
- Sample description: Programs of study by gender

Variables assessed in the study

The following variables were assessed in the study in the following order:

1. explicit trust in science
2. explicit utility of science and of personal experiences
3. implicit trust in science
4. implicit utility of science
5. epistemic beliefs
    - 26 items in five scales: certainty, simplicity, personal justification, justification by authority, and justification by multiple sources. The items for certainty and simplicity were translated from Bråten and Strømsø (2010). The items for the three justification scales were translated from Ferguson et al. (2013). Instead of a specific content domain, all items were phrased such that they referred to science in general.
6. knowledge about how science works
7. demographics
8. pretest for another study
    - prior beliefs for the following topics (6 items each)
        - Can regular physical exercise prevent cognitive problems at older ages?
        - Can a positive environment prevent adolescents from taking drugs?
        - Can sweeteners such as Aspartame harm health?
        - Does the consumption of violent media enhance the violent behavior of adolescents?
        - Do children need toys for their optimal development?
        - Is tourism beneficial for the Alps region?
    - interest in these topics (1 item each)

- description of 8 one-sentence scenarios with different targets (e.g., professor, best friend) who ask the participant to search for information
    - rating of perceived stakes in this situation
    - rating of the degree to which a personal relationship exists with this person
    - rating of the degree to which an academic relationship exists with this person
- rating of titles and authors for one of the six abovementioned topics in a fictitious search engine result page
    - relevance of the title to the question (20 titles to be rated)
    - authors' scientific expertise (16 authors to be rated)
    - authors' personal experiences (16 authors to be rated)

Questionnaire on utility of science and of personal experiences

The questionnaire on utility of science and of personal experiences was based on Kiemer and Kollar (2021). The items were rephrased to refer to science in general. The following items were used to assess the utility of science and of personal experiences (originally in German):

Table S1. Items of the questionnaire on utility of science and of personal experiences.

	German	English translation	Scale: utility of…
1	Die Erkenntnisse der Wissenschaft sind nützlich für persönliche Entscheidungen.	Scientific findings are useful for personal decisions.	science
2	Persönliche Entscheidungen sollten vor allem aufgrund eigener Erfahrungen oder Erfahrungen von anderen getroffen werden.	Personal decisions should be based mainly on own or others' experiences.	personal experiences
3	Wissenschaftliche Theorien und Befunde sind nützliche Werkzeuge, um persönliche Entscheidungen treffen zu können.	Scientific theories and findings are useful tools for making personal decisions.	science
4	Um eine gute persönliche Entscheidung treffen zu können, sind eigene Erfahrungen oder Erfahrungen von anderen unersetzlich.	For making a good personal decision, one's own or others' experiences are irreplaceable.	personal experiences
5	Wenn man die zentralen Erkenntnisse der Wissenschaft kennt, fällt es leichter, eine gute persönliche Entscheidung zu treffen.	If you know the central scientific findings, it is easier to make a good personal decision.	science
6	Der Rückgriff auf eigene Erfahrungen oder Erfahrungen von anderen ist wichtig, um gute persönliche Entscheidungen treffen zu können.	Falling back on one's own or others' experiences is important for making good personal decisions.	personal experiences
7	Eigene Erfahrung oder Erfahrung von anderen hilft, um gute persönliche Entscheidungen treffen zu können.	One's own or others' experiences can help a person make good personal decisions.	personal experiences
8	Wissen über zentrale Erkenntnisse der Wissenschaft ist von großer Bedeutung, um gute persönliche Entscheidungen treffen zu können.	Knowledge about central scientific findings is of great importance for making good personal decisions.	science

Knowledge about how science works

The following items were used to assess knowledge about how science works and were based on Retzbach et al. (2015). Only the items that were included in the final score are presented. The original items were in German and are presented at the end.

English translation of items

1. Please imagine the following situation: A scientist wants to check whether a certain drug is effective against depression. She has 400 patients available for her study who she splits up into two groups: 200 patients receive the drug from their family doctor, while the other 200 receive a similar looking pill without an active ingredient (a so-called placebo).

   From a scientific point of view, who should be informed about which patients received the drug and which the placebo? From among the following statements, please choose the one that you consider most likely to be true:

   - Neither the patients nor their family doctors should know which group the patients belong to; only the scientist has to know this for her analyses.
   - Only the patients have to be informed when they receive a pill without an active ingredient in order to obtain their informed consent. They must not be lied to. The family doctor does not need to know about it.
   - Only the family doctor of the patients should be informed about whether the patients received the actual drug or a placebo. Since the doctor knows his/her patients, he/she can better interpret the results and can give the scientist valuable hints for her analyses. The patients do not need to be informed.
   - Both the patients and their family doctor should be informed about it.

2. Please imagine the following situation: A provider of health courses wants to know whether it would be better to schedule the course "Yoga for beginners" in the morning, in the afternoon, or in the evening so that the largest number of people will participate. To answer this question, the provider plans a scientific study. There are different opinions about how the study should be conducted.

   From a scientific point of view, what is the best way to conduct the study? From among the following statements, please choose the one that you consider most likely to be true:

   - The study should be conducted for three years: In the first year, the course should be scheduled on Monday morning, in the second year on Tuesday afternoon, and in the third year on Friday evening. The provider can then analyze in which year the course had the most participants.
   - The study should be conducted for one year. From January to April, the course should be scheduled on Wednesday morning, from May to August on Wednesday afternoon, and from September to December on Wednesday evening. The provider can then analyze during which part of the year the course had the most participants.
   - The study should be conducted for one year. For the whole year, the course should be offered on Tuesday morning, Tuesday afternoon, and Tuesday evening. The provider can then analyze which course had the most participants.
   - The study should be conducted for three years: In the first year, the course should be scheduled on Tuesday morning, in the second year on Tuesday afternoon, and in the third year on Tuesday evening. The provider can then analyze in which year the course had the most participants.

3. Please imagine the following situation: Scientists want to know whether working in an open-plan office has a negative effect on productivity. Four scientists did a literature search in a scientific database.

Scientist A found a comprehensive case study showing that changing to working in an open-plan office had negative effects on a team at a big company.

Scientist B found a study on two teams at a big company in which one team changed to working in an open-plan office, while the other did not (control group design). This study also showed negative effects of working in an open-plan office.

Scientist C found a study on a team at a big company in which a phase involving single office work (pretest) was compared (pretest-posttest design) with a phase in which employees worked in an open-plan office (posttest). In this study, no negative effects of working in an open-plan office were found.

Scientist D found a meta-analysis in which several studies involving big companies with a control group design were compiled. In this meta-analysis, also no effects of working in an open-plan office were found.

From a scientific point of view, what can be said about negative effects of working in an open-plan office? From among the following statements, please choose the <u>one</u> that you consider most likely to be true:

- ○ Working in an open-plan office probably has negative effects because the case study found by scientist A proves this.
- ○ Working in an open-plan office probably has negative effects because the study with a control group design found by scientist B shows this.
- ○ Working in an open-plan office probably has no negative effects because, in the study with a pretest and posttest found by scientist C, no negative effects were found.
- ○ Working in an open-plan office probably has no negative effects because the meta-analysis found by scientist D did not show negative effects.

4. Please imagine the following situation: A clairvoyant claims that he can predict the courses of a set meal that an arbitrary guest will eat in a restaurant. It is known that the guest will choose an appetizer, a main dish, and a dessert. In this restaurant, 3 different appetizers, 7 different main dishes, and 5 different desserts are offered.

   What is the probability that the courses can be guessed by chance? From among the following statements, please choose the one that you consider most likely to be true:

   - o The probability is 1/3 because there are three different appetizers.
   - o The probability is 1/3 + 1/7 + 1/5 because the probabilities of the single courses have to be added.
   - o The probability is 1/7 because there are seven different main dishes.
   - o The probability is 1/3 * 1/7 * 1/5 because the probabilities of the single courses have to be multiplied.

5. Please imagine the following situation: A scientist found that within a radius of 500 meters around electrical towers, significantly more people get cancer than outside of this radius. He chose a level of significance of 5%.

   Which statement can be made? From among the following statements, please choose the one that you consider most likely to be true:

   - o Given that the same number of people get cancer within and outside of the radius, the probability of finding the significant result that he found is 5%.
   - o The probability of getting cancer within the radius is 95%.
   - o The probability of finding these results is 95% because the truth is that more people get cancer outside the radius.
   - o The probability of getting cancer outside the radius is 5%.

6. Please imagine the following situation: A scientist wants to publish a study he conducted. He writes an article about the study and submits it to a renowned scientific journal (with peer review).

   What happens to his article? From among the following statements, please choose the one that you consider most likely to be true:

   - The article goes to a proofreader working for the journal's publisher. The proofreader evaluates how well-written the article is and decides whether or not the article should be accepted.
   - The article goes to the editor of the journal. The editor evaluates the quality of the article and is the only person who gets to decide whether the article should be accepted.
   - The article is forwarded to several experts in the field who evaluate the quality of the article. Afterwards, the editor of the journal decides whether the article should be accepted.
   - The article goes to the editor of the journal and to a proofreader working for the journal's publisher. The editor evaluates the content of the article, the proofreader the language. Together they decide whether or not the article should be accepted.

7. Please imagine the following situation: Two scientists want to research whether people with similar personalities are more appealing to each other. Each scientist conducts a study with 1,000 participants who are each randomly placed into dyads with a person they did not know before.

In the study by scientist A, all participants have to fill out questionnaire alpha on their personality. Afterwards, they have to decide whether their partner is likeable or not.

In the study by scientist B, all participants also have to fill out a questionnaire on their personality, but it is questionnaire beta. Afterwards, they have to rate how likeable their partner is on a scale ranging from 1-10.

From a scientific point of view, did the two scientists conduct the same research? From among the following statements, please choose the <u>one</u> that you consider most likely to be true:

- The two scientists did not conduct the same research because they used different personality questionnaires.
- The two scientists conducted the same research; they only chose different ways to implement their question about the relationship between personality and likeability.
- The two scientists conducted the same research, but their results on the relationship between personality and likeability cannot be compared.
- The two scientists did not conduct the same research because they asked about likeability in different ways.

8. Please imagine the following situation: A scientist wants to research how sleep quality is influenced by light in the bedroom. Therefore, he examines 1,007 randomly chosen male students of medicine between the ages of 18 and 22. He finds that light in the bedroom has no negative effect on sleep quality.

From a scientific perspective and based on these findings, for which group of people can it be said that light in the bedroom does not negatively influence sleep quality? From among the following statements, please choose the one that you consider most likely to be true:

- For exactly the 1,007 students who participated.
- For all humans.
- For all students of medicine.
- For young male students of medicine.

Original German items

1. Stellen Sie sich bitte die folgende Situation vor: Eine Wissenschaftlerin möchte überprüfen, ob ein Medikament gegen Depression wirkt. Sie hat für ihre Untersuchung 400 Patienten zur Verfügung, die sie in zwei Gruppen aufteilt: 200 Patienten erhalten von ihrem Hausarzt das Medikament, die anderen 200 dagegen erhalten eine identisch aussehende, aber wirkstofflose Tablette (ein so genanntes Placebo).

   Wer sollte aus wissenschaftlicher Sicht darüber informiert sein, welche Patienten den Wirkstoff und welche das Placebo erhalten? Bitte kreuzen Sie aus den folgenden Aussagen eine an, die Ihrer Meinung nach am ehesten zutrifft:

   - Weder die Patienten noch ihre Hausärzte sollten wissen, zu welcher Gruppe sie gehören, nur die Wissenschaftlerin muss für ihre Auswertungen darüber Bescheid wissen.
   - Nur die Patienten müssen darüber informiert werden, wenn sie eine Tablette ohne Wirkstoff erhalten, um zuvor ihr Einverständnis einzuholen. Sie dürfen schließlich nicht belogen werden. Der Hausarzt muss nicht darüber Bescheid wissen.
   - Nur der Hausarzt der Patienten sollte darüber informiert sein, ob sie das Medikament oder ein Placebo erhalten. Da er seine Patienten kennt, kann er die Ergebnisse besser interpretieren und der Wissenschaftlerin wertvolle Hinweise für die Auswertung geben. Die Patienten müssen nicht darüber Bescheid wissen.
   - Sowohl die Patienten als auch der Hausarzt sollten darüber informiert sein.

2. Stellen Sie sich bitte folgende Situation vor: Ein Anbieter von Gesundheitskursen möchte wissen, ob der Kurs „Yoga für Einsteiger“ besser vormittags, nachmittags oder abends angeboten werden sollte, so dass möglichst viele Personen teilnehmen. Daher plant er eine wissenschaftliche Studie zu dieser Frage. Es gibt verschiedene Vorstellungen, wie diese Studie durchgeführt werden sollte.

   Welches ist aus wissenschaftlicher Perspektive die beste Vorgehensweise? Bitte kreuzen Sie aus den folgenden Aussagen eine an, die Ihrer Meinung nach am ehesten zutrifft:

   - Die Studie sollte über drei Jahre gehen: Im ersten Jahr wird der Kurs Montag vormittags angeboten, im zweiten Jahr Dienstag nachmittags, und im dritten Jahr Freitag abends. Es wird ausgewertet, in welchem Jahr am meisten Personen am Kurs teilgenommen haben.
   - Die Studie sollte ein Jahr lang durchgeführt werden. Der Kurs sollte von Januar bis April Mittwoch vormittags, von Mai bis August Mittwoch nachmittags und von September bis Dezember Mittwoch abends angeboten werden. Es wird ausgewertet, in welchem Zeitraum am meisten Personen am Kurs teilgenommen haben.
   - Die Studie sollte ein Jahr lang durchgeführt werden. Der Kurs sollte das ganze Jahr über Dienstag vormittags, Dienstag nachmittags und Dienstag abends durchgeführt werden. Es wird ausgewertet, an welchem Kurs am meisten Personen teilgenommen haben.
   - Die Studie sollte über drei Jahre gehen: Im ersten Jahr wird der Kurs Dienstag vormittags angeboten, im zweiten Jahr Dienstag nachmittags, und im dritten Jahr Dienstag abends. Es wird ausgewertet, in welchem Jahr am meisten Personen am Kurs teilgenommen haben.

3. Stellen Sie sich bitte die folgende Situation vor: Es geht um die Frage, ob sich Arbeiten im Großraumbüro negativ auf die Produktivität auswirkt. Vier Wissenschaftlerinnen recherchieren in einer wissenschaftlichen Datenbank nach Informationen dazu.

Wissenschaftlerin A hat eine ausführliche Fallstudie gefunden, die zeigt, welche negativen Effekte die Umstellung auf Arbeit im Großraumbüro auf ein Team eines großen Unternehmens hatte.

Wissenschaftlerin B hat eine Studie mit 2 Teams eines großen Unternehmens gefunden, in der ein Team auf Arbeit im Großraumbüro umgestellt wurde hat, das andere nicht (Kontrollgruppendesign). Diese Studie zeigte ebenfalls negative Effekte von Arbeit im Großraumbüro.

Wissenschaftlerin C hat eine Studie mit einem Team eines großen Unternehmens gefunden, in der eine Phase mit Einzelbüroarbeit (Vortest) mit einer Phase von Arbeit im Großraumbüro (Nachtest) verglichen wurde (Vor-Nachtest-Design). In dieser Studie zeigten sich keine negativen Auswirkungen von Großraumbüroarbeit.

Wissenschaftlerin D hat eine Metaanalyse gefunden, in der verschiedene Studien mit Kontrollgruppendesign in großen Unternehmen zusammengefasst wurden. In dieser Metaanalyse zeigten sich ebenfalls keine negativen Effekte von Arbeit im Großraumbüro.

Welche Aussage über die negativen Effekte von Arbeit im Großraumbüro kann aus wissenschaftlicher Perpektive gemacht werden? Bitte kreuzen Sie aus den folgenden Aussagen eine an, die Ihrer Meinung nach am ehesten zutrifft:

- Arbeit im Großraumbüro hat wahrscheinlich negative Effekte, da die Fallstudie von Wissenschaftlerin A dies belegt.
- Arbeit im Großraumbüro hat wahrscheinlich negative Effekte, da die Studie mit Kontrollgruppendesign von Wissenschaftlerin B dies zeigt.
- Arbeit im Großraumbüro hat wahrscheinlich keine negativen Effekte, da in der Studie mit Vor- und Nachtest von Wissenschafterlin C keine negativen Auswirkungen gefunden wurde.

- Arbeit im Großraumbüro hat wahrscheinlich keine negativen Effekte, da die Metaanalyse von Wissenschaftlerin D keine negativen Effekte nachweisen konnte.

4. Stellen Sie sich bitte die folgende Situation vor: Ein Hellseher behauptet, die Menüfolge eines beliebigen Gasts in einem Restaurant vorhersagen zu können. Es ist bekannt, dass der Gast eine Vorspeise, ein Hauptgericht und ein Dessert wählen wird. In diesem Restaurant gibt es 3 verschiedene Vorspeisen, 7 verschiedene Hauptgerichte und 5 verschiedene Desserts.

   Wie groß ist die Wahrscheinlichkeit, dass die Menüfolge zufällig richtig erraten wird? Bitte kreuzen Sie aus den folgenden Aussagen <u>eine</u> an, die Ihrer Meinung nach am ehesten zutrifft:

- Die Wahrscheinlichkeit ist 1/3, da es drei verschiedene Vorspeisen gibt.
- Die Wahrscheinlichkeit beträgt 1/3 + 1/7 + 1/5, da die Wahrscheinlichkeiten für die einzelnen Gänge addiert werden müssen.
- Die Wahrscheinlichkeit beträgt 1/7, da es sieben verschiedene Hauptgerichte gibt.
- Die Wahrscheinlichkeit beträgt 1/3 * 1/7 * 1/5, da die Wahrscheinlichkeiten für die einzelnen Gänge multipliziert werden müssen.

5. Stellen Sie sich bitte die folgende Situation vor: Ein Wissenschaftler hat herausgefunden, dass in einem Radius von 500 Meter um Strommasten signifikant mehr Menschen an Krebs erkranken als außerhalb dieses Radius. Das Signifikanzniveau hat er zuvor auf 5% festgelegt.

   Welche Aussage lässt sich treffen? Bitte kreuzen Sie aus den folgenden Aussagen <u>eine</u> an, die Ihrer Meinung nach am ehesten zutrifft:

- Die Wahrscheinlichkeit, dass diese Ergebnisse gefunden wurden, obwohl in Wahrheit innerhalb und außerhalb des Radius gleich viele Menschen an Krebs erkranken, liegt bei 5%.
- Die Wahrscheinlichkeit, innerhalb des Radius an Krebs zu erkranken, liegt bei 95%.

- o Die Wahrscheinlichkeit, dass diese Ergebnisse gefunden wurden, weil in Wahrheit außerhalb des Radius mehr Menschen an Krebs erkranken, liegt bei 95%.
- o Die Wahrscheinlichkeit, außerhalb des Radius an Krebs zu erkranken, liegt bei 5%.

6. Stellen Sie sich bitte die folgende Situation vor: Ein Wissenschaftler möchte eine Studie veröffentlichen, die er durchgeführt hat. Er schreibt einen Artikel über diese Studie und schickt ihn an eine renommierte Fachzeitschrift (mit Peer-Review-Verfahren).

   Was passiert mit seinem Artikel? Bitte kreuzen Sie aus den folgenden Aussagen <u>eine</u> an, die Ihrer Meinung nach am ehesten zutrifft:

- o Der Artikel geht an einen Lektor des Verlags der Zeitschrift. Der Lektor beurteilt, wie gut der Artikel geschrieben ist, und entscheidet darüber, ob der Artikel angenommen wird.
- o Der Artikel geht an den Herausgeber der Zeitschrift. Der Herausgeber beurteilt die Qualität des Artikels und entscheidet allein über die Annahme des Artikels.
- o Der Artikel wird an verschiedene Fachexperten weitergeleitet, die die Qualität des Artikels beurteilen. Danach entscheidet der Herausgeber der Zeitschrift über die Annahme des Artikels.
- o Der Artikel geht an den Herausgeber der Zeitschrift und an einen Lektor des Verlags der Zeitschrift. Der Herausgeber beurteilt den Artikel inhaltlich, der Lektor sprachlich. Gemeinsam entscheiden sie, ob der Artikel angenommen wird.

7. Stellen Sie sich bitte die folgende Situation vor: Zwei Wissenschaftlerinnen möchten untersuchen, ob sich Personen sympathischer sind, die eine ähnliche Persönlichkeit haben. Beide Wissenschaftlerinnen führen jeweils eine Studie mit jeweils 100 Personen durch, die zufällig in Paare aufgeteilt werden, die sich vorher noch nicht kannten.

   In der Studie von Wissenschaftlerin A müssen alle Teilnehmerinnen und Teilnehmer den Fragebogen Alpha zur Persönlichkeit ausfüllen. Anschließend müssen sie entscheiden, ob ihnen ihr Partner sympathisch oder unsympathisch ist.

   In der Studie von Wissenschaftlerin B müssen ebenfalls alle einen Fragebogen zu ihrer Persönlichkeit ausfüllen, allerdings den Fragebogen Beta. Danach müssen sie auf einer Skala von 1-10 ankreuzen, wie sympathisch ihnen ihr Partner ist.

   Inwiefern kann man aus wissenschaftlicher Perspektive sagen, dass die beiden Wissenschaftlerinnen dasselbe untersuchen? Bitte kreuzen Sie aus den folgenden Aussagen <u>eine</u> an, die Ihrer Meinung nach am ehesten zutrifft:

   - Die beiden Wissenschaftlerinnen untersuchen nicht dasselbe, da sie unterschiedliche Persönlichkeitsfragebögen verwenden.
   - Die beiden Wissenschaftlerinnen untersuchen dasselbe, sie haben nur unterschiedliche Formen gewählt, ihre Fragestellung zum Zusammenhang von Persönlichkeit und Sympathie umzusetzen.
   - Die beiden Wissenschaftlerinnen untersuchen dasselbe, allerdings kann man ihre Ergebnisse zum Zusammenhang von Persönlichkeit und Sympathie später nicht miteinander vergleichen.
   - Die beiden Wissenschaftlerinnen untersuchen nicht dasselbe, da sie Sympathie unterschiedlich erfragen.

8. Stellen Sie sich bitte die folgende Situation vor: Ein Wissenschaftler möchte untersuchen, wie die Schlafqualität von Licht im Schlafzimmer beeinflusst wird. Dazu untersucht er 1007 zufällig ausgewählte männliche Medizin-Studierende im Alter von 18-22 Jahren. Er findet, dass Licht im Schlafzimmer keinen negativen Effekt auf die Schlafqualität hat.

Für welche Gruppe von Menschen kann man auf der Basis dieser Ergebnisse aus wissenschaftlicher Perspektive die Aussage treffen, dass Licht im Schlafzimmer die Schlafqualität nicht negativ beeinflusst? Bitte kreuzen Sie aus den folgenden Aussagen eine an, die Ihrer Meinung nach am ehesten zutrifft:

- Nur für genau die 1007 Studierenden, die teilgenommen haben.
- Für alle Menschen.
- Für alle Medizinstudierenden.
- Für junge männliche Medizinstudierende.

Sample: Programs of study by gender

In Table S2, the participants' study program are listed by gender.

Table S2. Programs of study by gender.

Program of study	Female	Male	Overall
Teacher education	40	7	48
**Master's program**			
Psychology	24	2	26
Educational science	13	1	14
Sociology	1	3	4
Political science	1	2	3
Communication science	2	0	2
Economics	2	3	5
Computer sciences	2	0	2
Other humanities/cultural sciences	5	4	9
Other Master's program	0	1	1
**Bachelor's program**			
Psychology	43	5	48
Educational science	22	5	27
Sociology	10	3	13
Political science	1	6	7
Communication science	22	5	27
Economics	3	4	7
Computer sciences	2	3	5
Other humanities/cultural sciences	5	2	7

Note. Discrepancies between the sums of the female and male students and the overall number of students per program are due to missing or third gender category answers on the gender variable.

References

Bråten, I., & Strømsø, H. I. (2010). Effects of task instruction and personal epistemology on the understanding of multiple texts about climate change. *Discourse Processes, 47*(1), 1-31. https://doi.org/10.1080/01638530902959646

Ferguson, L. E., Bråten, I., Strømsø, H. I., & Anmarkrud, Ø. (2013). Epistemic beliefs and comprehension in the context of reading multiple documents: Examining the role of conflict. *International Journal of Educational Research, 62*, 100-114. https://doi.org/10.1016/j.ijer.2013.07.001

Kiemer, K., & Kollar, I. (2021). Source selection and source use as a basis for evidence-informed teaching. *Zeitschrift für Pädagogische Psychologie, 35*(2-3), 127-141. https://doi.org/10.1024/1010-0652/a000302

Retzbach, J., Otto, L., & Maier, M. (2015). Measuring the perceived uncertainty of scientific evidence and its relationship to engagement with science. *Public Understanding of Science, 25*(6), 638-655. https://doi.org/10.1177/0963662515575253
